# Alternative Splicing of the RAGE Cytoplasmic Domain Regulates Cell Signaling and Function

**DOI:** 10.1371/journal.pone.0078267

**Published:** 2013-11-08

**Authors:** Joel Jules, Dony Maiguel, Barry I. Hudson

**Affiliations:** 1 Division of Endocrinology, Diabetes & Metabolism, Leonard M. Miller School of Medicine, University of Miami, Miami, Florida, United States of America; 2 Division of Nephrology and Hypertension, Leonard M. Miller School of Medicine, University of Miami, Miami, Florida, United States of America; University of Pittsburgh, United States of America

## Abstract

The Receptor for Advanced Glycation End-products (RAGE) is a multi-ligand receptor present on most cell types. Upregulation of RAGE is seen in a number of pathological states including, inflammatory and vascular disease, dementia, diabetes and various cancers. We previously demonstrated that alternative splicing of the RAGE gene is an important mechanism which regulates RAGE signaling through the production of soluble ligand decoy isoforms. However, no studies have identified any alternative splice variants within the intracellular region of RAGE, a region critical for RAGE signaling. Herein, we have cloned and characterized a novel splice variant of RAGE that has a truncated intracellular domain (RAGEΔICD). RAGEΔICD is prevalent in both human and mouse tissues including lung, brain, heart and kidney. Expression of RAGEΔICD in C6 glioma cells impaired RAGE-ligand induced signaling through various MAP kinase pathways including ERK1/2, p38 and SAPK/JNK. Moreover, RAGEΔICD significantly affected tumor cell properties through altering cell migration, invasion, adhesion and viability in C6 glioma cells. Furthermore, C6 glioma cells expressing RAGEΔICD exhibited drastic inhibition on tumorigenesis in soft agar assays. Taken together, these data indicate that RAGEΔICD represents a novel endogenous mechanism to regulate RAGE signaling. Significantly, RAGEΔICD could play an important role in RAGE related disease states through down regulation of RAGE signaling.

## Introduction

The Receptor for Advanced Glycation End-products (RAGE) is a transmembrane protein member of the immunoglobulin superfamily which has been demonstrated to be involved in a number of important biological processes including cell migration, invasion, viability, and apoptosis [1], [2]. Through modulating these processes, RAGE has been implicated in various pathological disease states including diabetic vascular disease, inflammatory disease and cancer [3], [4]. RAGE possesses multi-ligand binding affinity for a wide range of molecules including Advanced Glycation End-products (AGEs), various S100/calgranulins (S100A4, A6-8, S100B and S100P) [2], [4], [5], and the high mobility group box-1 (HMGB1) protein [6] amongst others. Notably, RAGE and its ligands are highly upregulated in tumorigenic state, and their increased expression correlate with higher histological grades in human samples of various cancers [4], [7]–[12]. Ligand binding to RAGE can activate a diverse range of cellular signaling pathways including various mitogen activated protein kinase (MAP) kinases (ERK 1/2, p38, SAPK/JNK) and Rho GTPases (Rac1, Cdc42), which subsequently lead to activation of various transcription factors including NF-kB and SP-1 [1], [13]–[18]. Activation of RAGE-mediated signaling results in the induction of cellular pathways and properties associated with tumor invasion and metastasis. These cellular properties include increased cell migration, proliferation, cellular survival and invasion of the extracellular matrix [1], [2].

The mature RAGE protein is organized into three main domains: an extracellular domain (ECD) containing the ligand binding site, a short transmembrane region, and a cytoplasmic intracellular domain (ICD) [13], [14], [19]. The RAGE ICD has been shown to be essential for ligand-induced RAGE activation, as truncation of this domain imparts a dominant negative (DN) effect on RAGE function [1], [13], [18], [20]. This DN effect impairs receptor activation by blocking RAGE-ligand signaling and subsequently impacting cell properties such as migration, proliferation, adhesion and invasion [1], [13], [18], [20]. Whilst the mechanism by which the RAGE ICD transmits signaling is not completely clear, many proteins, including diaphanous-1, ERK1/2, PKCζ, TIRAP and DOCK7 [13], [21]–[23], have been shown to interact with RAGE. Therefore, a better understanding how RAGE ICD is capable of mediating this diverse array of cell signaling and downstream effects is clearly needed.

One particular mechanism cell surface receptors utilize to regulate their signaling cascades is the alternative splicing of their ICD. This is common among members of the immunoglobulin superfamily, to which RAGE belongs [24], [25]. To address this, ours and other groups have extensively characterized the alternative splicing of RAGE to identify RAGE regulatory mechanisms [26]–[33]. However, the majority of RAGE splice variants identified to-date only affect either the ligand binding site or result in the production of soluble RAGE isoforms [26]–[33]. No studies to date have identified any splice variants that affect the RAGE ICD. Hence, we have described a novel RAGE variant that has a truncated ICD (RAGEΔICD). RAGEΔICD is prevalent at the transcript level in both human and murine tissues. Functional cell studies by overexpression indicate that RAGEΔICD displays a dominant negative function on RAGE cell signaling and effects. We thus propose that RAGEΔICD acts as an endogenous mechanism to regulate RAGE signaling. This finding adds to the diversity of RAGE signaling and function in biology and disease.

## Materials and Methods

### Splice Variant Cloning and Identification

To identify splice variation within the RAGE ICD, full length RAGE for both human and mouse were amplified by PCR using lung cDNA from the Multiple Tissue cDNA Panel (Clontech Laboratories) as previously described [30], [31]. The PCR product was then cloned into the TOPO TA vector (Invitrogen), and 20 clones were selected for each human and mouse RAGE. The C-terminal region of RAGE from plasmid DNA was sequenced with primers covering the transmembrane and ICD region of RAGE (Human RAGE exon 8 forward: 5′-TCAGGACCAGGGAACCTACA-3′; Human RAGE 3′UTR reverse: 5′GTCTGAGGCCAGAACAGTTC-3′. Mouse RAGE exon 8 forward: 5′- GATGAGGGCACCTATAGCTG-3′; Mouse RAGE 3′UTR reverse: 5′-GGATGGAATGTGGGGGAG-3′).

To assess the relative prevalence of the RAGE ICD variant (RAGEΔICD) identified above, PCR- restriction digest analysis was performed as previously described [30], [31]. In brief, full length RAGE for both human and mouse were amplified from the cDNA panel for lung, heart, kidney and brain as described above and cloned. 50 colonies were selected for each human/mouse from each tissue and PCR performed using the exon 8–3′UTR primers. The PCR product was then digested overnight at 37°C with 10 U of HpyAV (mouse) or HpyAV and BamHI (human), and then electrophoresed for 2h on a 3% agarose gel (Invitrogen) at 100 V. A relative percentage of distribution was then calculated. PCRs from colonies of different patterns from the predicted RAGE full-length sequence were repeated to verify data. Where different patterns were seen, plasmid DNA was sequenced. For both human and mouse RAGEΔICD, DNA sequences were submitted to Genbank (human accession number KC692917; mouse accession number KC692918).

### Cell Culture and Transfection

Rat C6 glioma cells were purchased from American Type Culture Collection (ATCC) and maintained in Dulbecco's Modified Eagle Medium (DMEM) supplemented with 10% fetal bovine serum (FBS) (Atlanta Biologicals). Full-length RAGE and the RAGE ICD variant were subcloned from the TOPO TA cloning vector to pcDNA3.1 vector (Invitrogen). C6 cells were transfected with RAGE, RAGEΔICD and empty vector control (mock) using Fugene 6 (Promega), and stably expressing cells were generated by selection with G418. RAGE/RAGEΔICD expression was verified by Western blot as described below.

### Cell Surface Biotinylation Assay

Cell surface biotinylation was performed using the Pierce Cell Surface Protein Isolation Kit according to manufacturer's instructions (Rockford, IL). Briefly, 2×10^6^ C6 cells (RAGE/RAGEΔICD/vector) were washed with ice-cold phosphate buffered saline (PBS), followed by incubation with Sulfo-NHS- SS-Biotin solution for 1 hour at 4°C. Cell surface biotinylation was stopped with Quenching solution. Cells were collected and washed with Tris-buffered saline (TBS) before lysing. Supernatant was collected and mixed 1∶1 with NeutrAvidin Agarose. Samples were mixed and incubated overnight at 4°C, pelleted by centrifugation, washed and finally eluted in SDS-PAGE Sample Buffer containing DTT and stored at −80°C until usage. Samples were analyzed by Western blot with anti-RAGE polyclonal antibody. Input lysate was used to determine equal loading by Western blot. The assay was repeated independently three times.

### Flow Cytometric Analysis/Cell Apoptosis Assay

Cell apoptosis assay was performed using the Annexin V apoptosis kit (Invitrogen) as described [2]. Apoptosis was quantified using a FACS Caliber flow cytometer (BD Biosciences). For RAGE cell surface assays, 2×10^5^ cells suspended in DMEM with 10% of FBS were incubated with 1 µg of rabbit RAGE polyclonal antibody (H300, Santa Cruz Biotechnologies) or mouse IgG istotype control antibody on ice for 1 hour. Cells were centrifuged at 900 rpm for 5 minutes and washed three times with DMEM followed by centrifugation at 900 rpm for 5 minutes. Cells were then incubated with secondary antibodies (rabbit Alexa-647 secondary antibody for RAGE or mouse Alexa-647 secondary antibody for istotype control) on ice for 30 minutes, followed by washing and centrifugation at 900 rpm for 5 minutes. Finally, cells were suspended in 0.2 ml of DMEM for immediate analysis using a FACS Caliber flow cytometer (BD Biosciences). The experiment was repeated independently three times.

### Cell Signaling and Western Blot Analysis

Stably expressing C6 cells (RAGE and RAGEΔICD) were seeded at 2×10^5^ cells per well of a 6-well dish and grown for 24 hours. Cells were starved overnight in serum-free DMEM. Cells were incubated with/without 5 µg/ml S100B (Calbiochem) for 5 minutes at 37°C to stimulate RAGE signaling as previously described [2], [13]. Cells were then placed on ice and washed in ice cold PBS. Cell lysates were prepared by performing cell lysis with MPER buffer (Pierce) containing protease and phosphatase Cell prepared by performing cell lysis with MPER buffer (Pierce) containing protease and phosphatase inhibitors (Sigma). 10 µg protein samples were electrophoresed using the NuPAGE Gel Electrophoresis System (Invitrogen) and transferred to Immobilon-FL PVDF (Millipore) membranes. Membranes were blocked with Odyssey Blocking Buffer (Licor) and incubated with primary antibody overnight at 4°C. Blots were washed with T-BST and incubated with either IRDye 680LT Goat anti-mouse or Goat anti-rabbit for 1 h, washed four times in T-BST, and visualized using an Odyssey IR Imaging System (LiCor). Antibodies used were against RAGE (H300, Santa Cruz), phospho-p44/42ERK (9101s), phospho-JNK (9251s), phospho-p38 (9211s) were obtained from Cell Technology, Inc. (Beverly, MA). Anti GAPDH (MAB374) and β-actin (MAB1501) antibodies were obtained from Millipore. Antibodies were all used according to manufacturer's instructions. The experiment was repeated independently three times.

### Soft Agar Assay

Anchorage independent soft agar assays were carried as previously described [34]. 4×10^3^ cells per well of 96-well plate were seeded in a layer of 0.4% agarose in Iscove's Modified Dulbecco's Medium (IMDM) with 10% FBS and 0.2 mg/ml G418 and cultured for 14 days. Tumor colonies were stained on 14 days with 0.05% crystal violet in 10% ethanol at room temperature. Images were acquired using a Nikon Eclipse TS100 microscope. The assay was performed in triplicate and repeated independently three times.

### Cell Migration and Invasion assays

Cell migration assays were performed using transwell migration chambers as previously described [2]. Cells (5×10^3^) were seeded in 8-µm porous transwell chambers (ThinCerts, Greiner) in serum-free DMEM, and incubated in 24 well plates with either 5 µg/ml of S100B or DMEM supplemented with 1% FBS as a chemoattractant for 24 hours. For invasion assays, the upper chamber was coated with 12.5 µg of Growth Factor Reduced Matrigel (BD Biosciences) as previously described [35]. Following incubation, migration/invaded cells were fixed with methanol for 10 minutes and stained with 2% crystal violet in 2% ethanol solution. Non-migrated cells were removed with a cotton swap. To quantify migration, the cell stain was extracted with 10% acetic acid and measured at 595 nm using an iMark Microplate Reader (Biorad). To calculate the invasive potential of cells, an “invasive index” was calculated as previously described [35], where: invasive index  =  the number of invaded cells/number of migrated cells ×100.

### Cell Adhesion Assay

Cells were seeded at 2×10^4^ in serum-free media on 12-well plate and incubated at 37°C for 2 hours. Following the incubation period, cells were washed twice with PBS to remove non-adherent cells, and fixed with 4% paraformaldehyde for 15 minutes at room temperature. Attached cells were stained with 0.1% crystal violet in 10% ethanol for 2 hours at room temperature. Cell stain was extracted and quantified as above. The assay was performed in triplicates and repeated independently at three times.

### Cell proliferation assay

Cell proliferation assays were performed using the AlamarBlue Assay Kit (Invitrogen) according to the manufacturer's instructions. Cells were seeded into 96 well plates and incubated for 4 hours before the addition of AlamarBlue Solution. Cells were then incubated for 48 hours in media supplement with 1% FBS. Fluorescence was measured as above and data normalized by a control medium well with no added cells. The assay was performed in duplicates and repeated independently five times.

### Statistical Analysis

All statistical analyses were performed using GraphPad Prism version 6.00 for Windows, (GraphPad Software, San Diego California USA). Student's t-test was used for comparison between groups and a probability value of p<0.05 was considered to be of significance.

## Results

### Identification of a RAGE cytoplasmic domain splice variant

**Figure 1:**
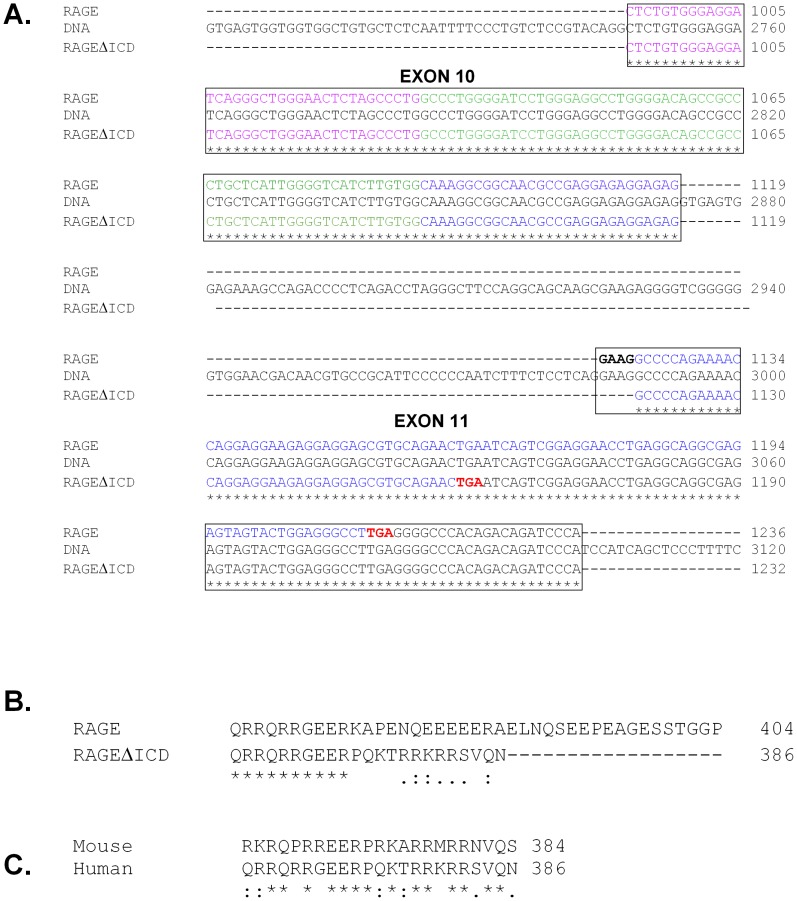
Alternative splicing of RAGE results in a truncated cytoplasmic domain conserved amongst humans and mice. **A.** RAGEΔICD is formed through alternative splicing of the 5′ end of exon 11 of RAGE. The DNA and cDNA sequences of human RAGE were aligned by ClustalW. The alternative 3′ acceptor site at the beginning of exon 11 is shown in bold. Stop codons for canonical RAGE and the novel RAGEΔICD protein reading frames are shown in red. The regions of RAGE and RAGEΔICD encoding the extracellular domain (purple), transmembrane (green) and intracellular domain (blue) are shown. **B**. Alignment of the protein sequence of the cytoplasmic domains of human RAGE and human RAGEΔICD. Conserved amino acids are shown by a “*”, amino acids with strong similar properties are shown by “:” and those with weak similar properties by a “.” Total amino acid number of each isoform is indicated to the right of the protein sequence.

Data from multiple groups have demonstrated that the RAGE gene undergoes extensive alternative splicing in humans, mice and other species [26]–[33]. These include variants of the extracellular domain with alterations in the ligand binding domains, or truncated variants that produce secreted soluble isoforms of the receptor [36]. To-date, no studies have identified any variants that alter the intracellular domain (ICD). Cloning and sequencing of the RAGE cDNA region encoding the ICD revealed a common splice variant that lacked 4 base pairs (GAAG) of the 5′ end of exon 11 resulting from an alternative 3′ acceptor site (**Fig. 1A**). Bioinformatic analysis revealed that this splice variation shifts the reading frame in the ICD of RAGE at amino acid 375, and subsequently resulted in a premature stop codon (**Fig. 1**). This splicing change resulted in a RAGE protein variant that contained the extracellular and transmembrane domain of RAGE, but with a much shorter ICD than canonical RAGE (**Fig. 1B**). The ICD of RAGEΔICD contained the first 10 amino acids as RAGE, followed by 13 unique amino acids, and an overall truncation of 18 amino acids. Cloning and sequencing of mouse cDNA revealed that the splice variant (GAAG removal at the 5′ end of exon 11) is conserved between mouse and human. Furthermore, this protein coding change in the RAGE ICD was found to be identical between human and mouse (**Fig. 1C**). As per our previous studies classifying RAGE splice variants [30], [31], we have termed this novel RAGE splice variant “RAGE splice variant 20.” But, for clarity herein we have referred to it as RAGEΔICD (RAGE deletion of the intracellular domain).

### RAGEΔICD is widely expressed in humans and mice

To investigate the distribution of RAGEΔICD in tissue and across different species we performed high throughput analysis as previously used for screening of RAGE splice variants [30], [31]. Primers were designed to amplify from exon 8 to the 3′ UTR for both human and mouse RAGE cDNA to cover the region of alternative splicing of the RAGE ICD (**Fig. S1&2**). Tissue cDNA was amplified by PCR, and cloned, and 50 clones per tissue was screened by restriction digestion to allow the identification of the 4 bp splicing change (**Fig. S1&2**). Assessment of the digestion pattern gave a similar distribution of RAGEΔICD across tissues and species (**Table 1** **&** **2**), with RAGEΔICD being more prevalent in mice. In both species, relative abundance across tissues was of a similar order with kidney > heart > brain > lung. Interestingly, in murine tissue RAGEΔICD was the most prevalent splice variant in kidney, accounting for 86% of all variants. In addition, this method of screening allowed the detection of other RAGE splice variants within the exon 8 to 3′UTR region. Similar distributions of RAGE splice variants from humans and mice were comparable those reported in prior studies [30], [31].

**Table 1:** Prevalence of human RAGEΔICD in lung, kidney, heart and brain.

	Lung	Kidney	Heart	Brain
**RAGE**	80% (40)	82% (41)	82% (41)	90% (45)
**RAGE_v1**	18% (9)	14% (7)	14% (7)	8% (4)
**RAGEΔICD (RAGE_v20)**	2% (1)	4% (2)	4% (2)	2% (1)

The percent frequency of splice variant detected is shown (with total number detected) which was obtained by restriction digest screening of RAGE exon 8-3UTR colony PCR.

**Table 2:** Prevalence of mouse RAGEΔICD in lung, kidney, heart and brain.

	Lung	Kidney	Heart	Brain
**mRAGE**	60% (30)	4% (2)	46% (23)	44% (22)
**mRAGE_v1**		2% (1)	2% (1)	4% (2)
**mRAGE_v4**	36% (18)	6% (3)	38% (19)	32% (16)
**mRAGE_v3/v5**		2% (1)	2% (1)	2% (1)
**RAGEΔICD (mRAGE_v20)**	4% (2)	86% (43)	12% (6)	18% (9)

The percent frequency of splice variant detected is shown (with total number detected) which was obtained by restriction digest screening of RAGE exon 8-3UTR colony PCR.

### RAGEΔICD affects RAGE ligand signaling

**Figure 2:**
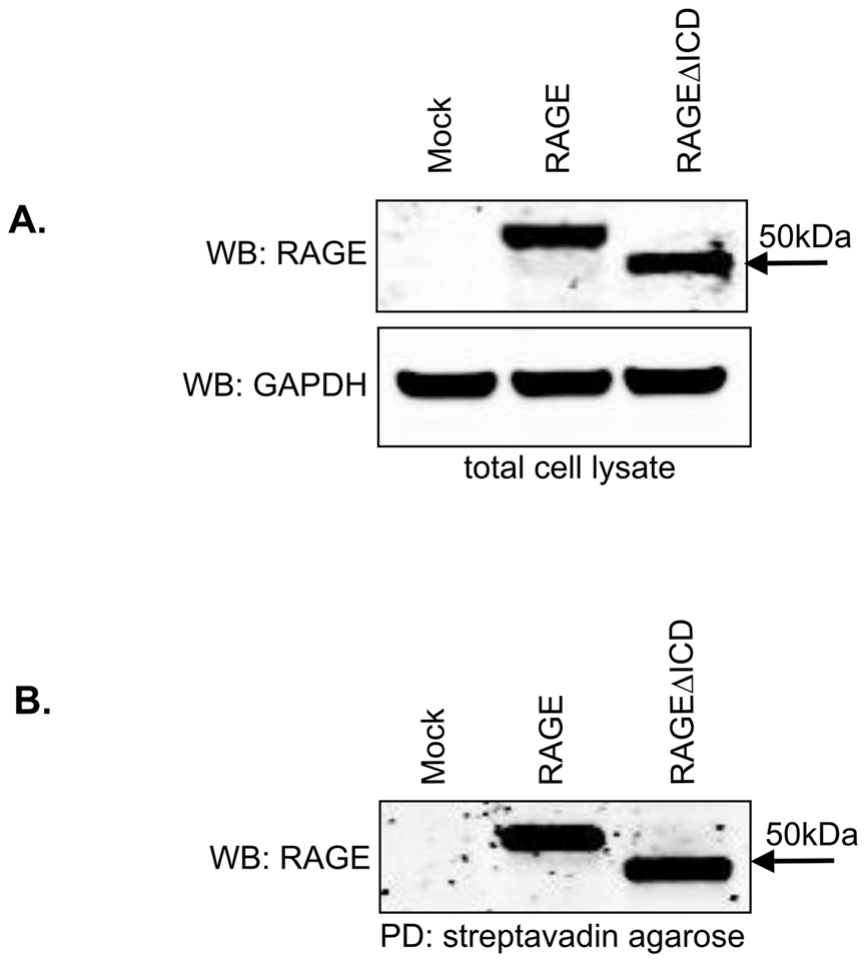
RAGEΔICD is expressed in cells and is present on the cell surface. **A–B.** Cell surface biotinylation was performed with C6 glioma cells expressing RAGE, RAGEΔICD, or empty vector (mock). Western blot for RAGE using polyclonal anti-RAGE antibodies was performed using total input of cell lysate (A) and extracts subjected to cell surface biotinylation which was followed by pull-down with streptavidin agarose (B). GAPDH was detected in total cell lysate by western blot using monoclonal anti-GAPDH for normalization.

Previous studies have shown an essential role for the RAGE ICD in cell signaling. Deletion of the ICD of RAGE imparts a dominant negative effect (DN-RAGE) by blocking key signaling pathways downstream of RAGE. Also, DN-RAGE through abrogating RAGE signaling is capable of preventing RAGE driven tumorigenesis and vascular disease in animal models [1], [13], [18], [20]. As a first test of whether RAGEΔICD might represent an endogenous DN-RAGE, we investigated its effect on RAGE signaling in tumor cells. C6 glioma cells were generated that stably expressed RAGE, RAGEΔICD or empty vector control (mock). The C6 cell line is both an established model for RAGE signaling and tumorigenesis in vitro and in vivo [2], [13], [37]. Western blot analysis confirmed the expression of both RAGE and RAGEΔICD in the C6 cells (**Fig. 2A**). To confirm that RAGEΔICD was present on the cell surface, we performed cell surface biotinylation studies (**Fig. 2B**). The results showed that both total and cell surface levels of RAGE and RAGEΔICD were expressed at a similar level. Data was confirmed by flow cytometry (data not shown).

**Figure 3:**
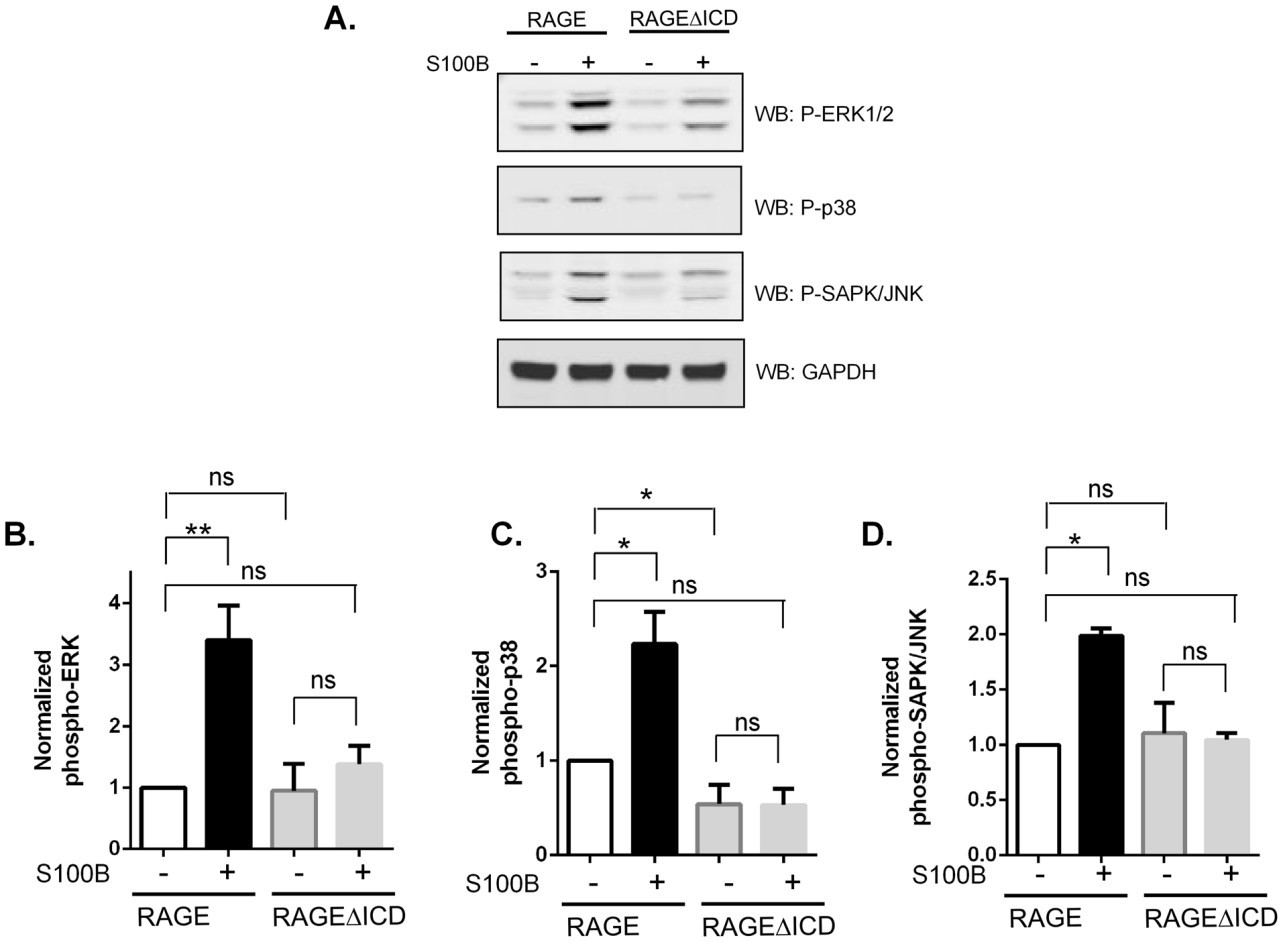
RAGEΔICD affects RAGE-ligand signaling. **A.** C6 glioma cells expressing RAGE or RAGEΔICD were stimulated with 5 ug/ml S100B for 5 minutes. Cell lysate was subjected to Western blot for antibodies against phospho-ERK1/2, p38 and SAPK/JNK. GAPDH was detected in total cell lysate by Western blot using monoclonal anti-GAPDH for normalization. **B–D**. Quantification of triplicate repeats normalized to GAPDH was performed for pERK (B), p38 (C), and pJNK, where *, p<0.05 and **, p = 0.005.

RAGE can induce various signaling pathways; however, the most characterized of these signaling pathways is the MAP kinase pathway [1], [2]. To determine whether the RAGEΔICD splice variant affected RAGE ligand signaling, C6-RAGE and RAGEΔICD expressing cells were stimulated with the RAGE ligand S100B. Whereas activation of RAGE expressing cells by S100B resulted in a striking activation of ERK1/2, p38 and SAPK/JNK, the RAGEΔICD expressers did not induce significant activation of these pathways in response to S100B (Fig. 3). Data suggest that RAGEΔICD may act as a DN-RAGE isoform by altering MAP kinase signaling. To further probe this issue, we investigated the impact of RAGEΔICD on cell functions.

### RAGEΔICD reduces cell motility and invasiveness

**Figure 4:**
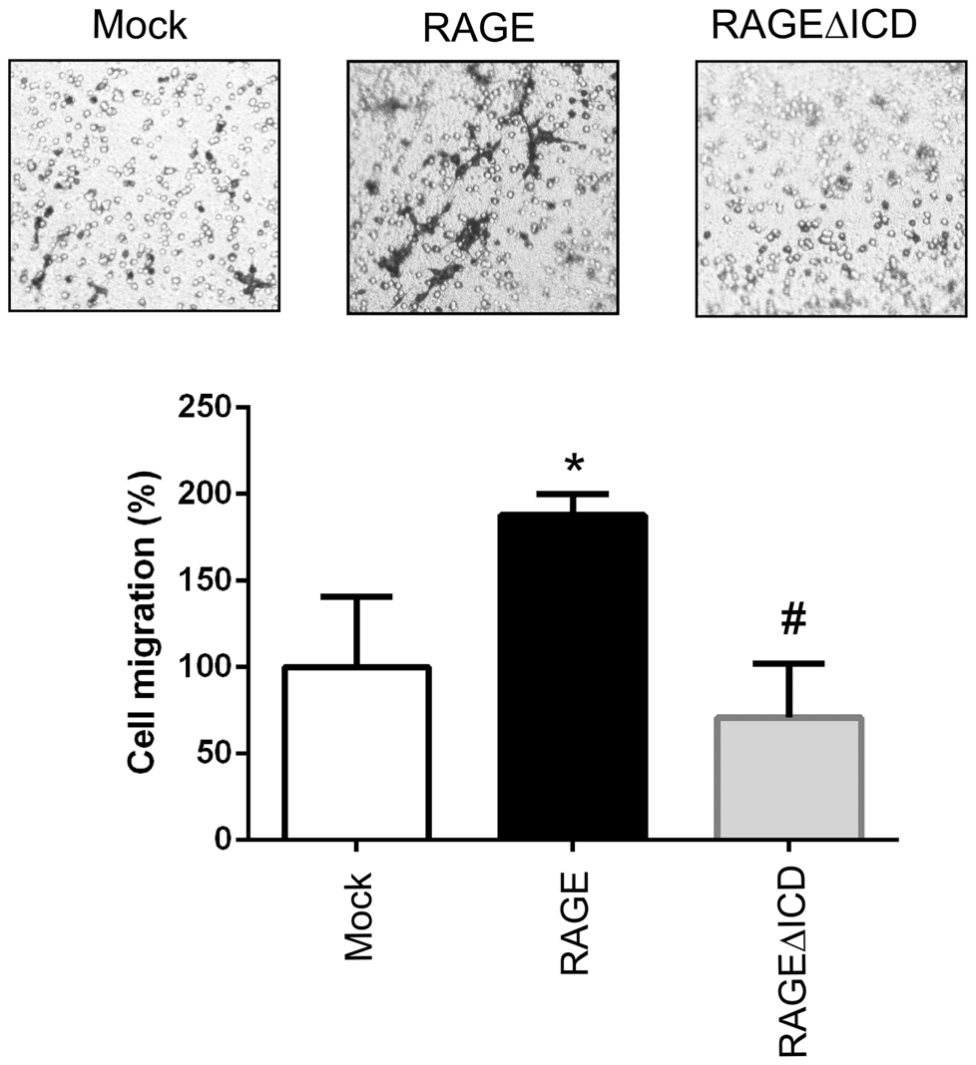
RAGEΔICD affects S100B induced cellular migration. **A–B.** C6 glioma cells expressing RAGE, RAGEΔICD, or empty vector (mock) were assessed for S100B induced migration using chemotaxis assays. Cells were seeded into the upper chamber of a Boyden transwell filters and allowed to migrate toward 5 ug/ml S100B stimulant for 24 hours. **A**. Representative images are shown. **B.** quantitation of relative cell migration normalized to mock transfected cells. Data are means ± SEM from three independent experiments, where *, significant differences (P = 0.03) between groups; and #, p = 0.006.

**Figure 5:**
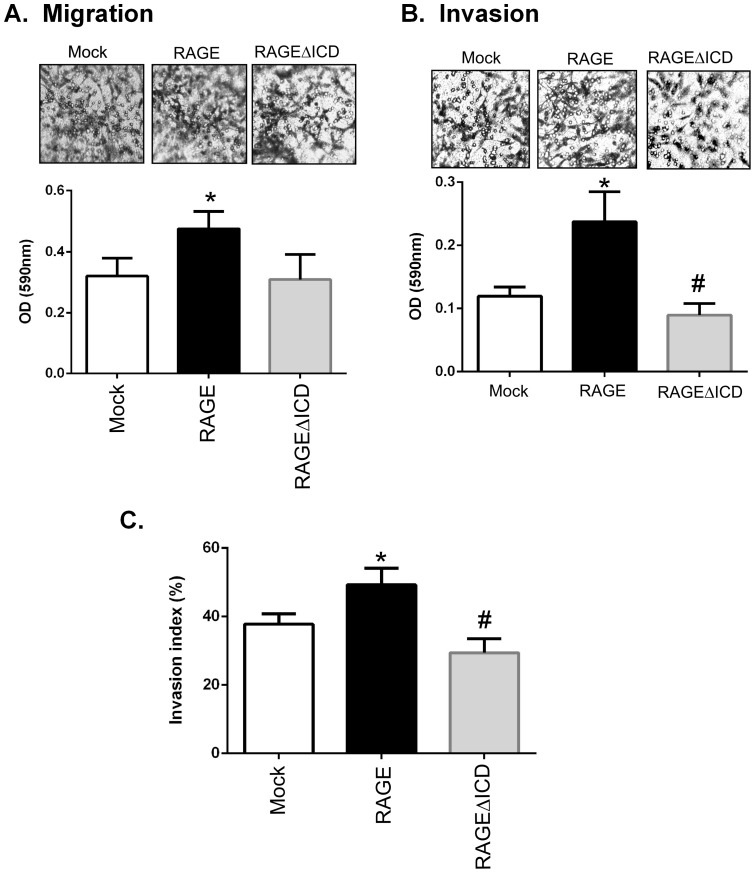
RAGEΔICD affects cellular migration and invasion. **A–C.** C6 glioma cells expressing RAGE, RAGEΔICD, or empty vector (mock) were assessed for cell migration (A) and cell invasion (**B**) using chemotaxis assays. Cells were seeded into the upper chamber of a Boyden transwell filters and allowed to migrate toward 1% FBS stimulant for 24 hours. For invasion assays, transwell filters were coated with 12.5 ug of Matrigel. **C**. To assess the “invasive index” of cells, the ratio of migrated cells by invaded cells was calculated and expressed as a percentage. Data are means ± SEM from three independent experiments. *, significant differences (P≤0.05) between mock and RAGE, whereas # significant differences (P≤0.05) between RAGE and RAGEΔICD.

Next, we examined the underlying cellular mechanisms by which RAGEΔICD might impact C6 functions. Because RAGE plays important roles in cell migration and invasion, we first examined the role of RAGEΔICD in these cellular processes. First, we examined cell migration via transwell migration assays, in which cells were allowed to move through a porous membrane in response to S100B (used as a chemoattractant). C6 glioma cells expressing RAGE displayed increased s100B stimulated cell migration compared to mock transfected cells (1.9 fold increase, p = 0.03) (**Fig. 4**). In contrast, cells expressing RAGEΔICD had decreased cell migration compared to RAGE expressing cells (p = 0.005) but displayed a similar migratory state compared to mock transfected cells (**Fig. 4**). As RAGE signaling and cellular induced effects can be induced by multiple ligands, we also explored the role of a broad physiological stimuli (serum) as used in many RAGE studies [38] [39] [40] [41] [42] [43]. As we wanted to see the broad biological effects of RAGEΔICD, we used serum which represents a rich source of numerous RAGE ligands [44] [45] [46]. Transwell migration was then performed using 1% FBS as a broad chemoattractant to assess the role of RAGEΔICD on cell physiology. Cells expressing RAGE strongly promoted cell migration compared to the mock expressers (1.5 fold increase, p<0.05) (**Fig. 5A**). However, the RAGEΔICD expressers had decreased cell migration compared to the RAGE expressers (p<0.05) but displayed a similar migratory state compared to the mock transfected cells (**Fig. 5A**).

To investigate whether RAGEΔICD impacted tumor cell invasion, we performed chemoinvasion assays using transwell migration chambers as mentioned above, but these chambers were coated with extracellular matrix (ECM). In cells expressing RAGE, chemoinvasion was increased 2-fold (p>0.05) compared to mock expressing cells in response to 1% FBS chemoattractant (Fig. 5B). In contrast, cell invasion was significantly decreased in RAGEΔICD expressing cells as compared to both RAGE and mock expressing cells (p>0.05). To distinguish the invasive effect from cell movement, we examined the ratio between the invaded/migrated cells to obtain an “invasive index” [35], [47]. Compared to mock, the RAGE expressing cells exhibited higher invasive index (p<0.05) (**Fig. 5C**). In contrast, RAGEΔICD had lower invasive index than both the RAGE and mock expressers (**Fig. 5C**). These data together demonstrate a clear role for RAGEΔICD in cell migration and invasion.

### RAGEΔICD affects cell adhesion and viability

**Figure 6:**
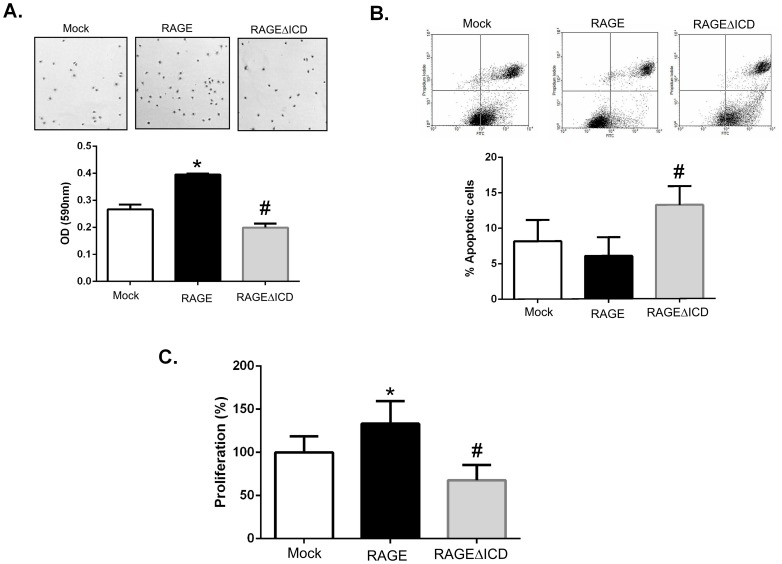
RAGEΔICD alters cell adhesion and viability. **A.** Cell adhesion assays were performed by seeding C6 glioma cells expressing RAGE, RAGEΔICD, or empty vector (mock) onto cell culture dishes for 2 hours. Adhered cells were fixed and stained with crystal violet before quantification by a spectrophotometer. **B**. Apoptosis was assessed by staining C6 glioma cells expressing RAGE, RAGEΔICD, or empty vector (mock) for Annexin V (X-axis) and PI (Y-axis) which as followed by flow cytometric analysis. **C**. Proliferation was assessed using the Alamar Blue reagent and assessed after 48 hours of culture of C6 glioma cells expressing RAGE, RAGEΔICD, or empty vector (mock). Data are means ± SEM from three independent experiments. *, significant differences (P≤0.05) between mock and RAGE, whereas # significant differences (P≤0.05) between RAGE and RAGEΔICD.

We next sought to determine whether RAGEΔICD affected other tumorigenic cell properties such as adhesion, proliferation and viability. Our adhesion assays revealed that RAGE expressing cells were more adhesive than C6-mock cells to tissue culture plates under basal conditions, whereas C6-RAGEΔICD cells displayed less adhesion compared to both the RAGE and mock expressers (**Fig. 6A**). Moreover, analysis of apoptosis under basal conditions by flow cytometry using Annexin V/PI staining revealed that C6-RAGE cells had lower levels, although not statistically significant, of apoptotic cells than C6-mock (6% vs 8%) (**Fig. 6B**). However, the RAGEΔICD expressers displayed significantly higher levels of cellular apoptosis (13%) than both mock and RAGE expressers (**Fig. 6B**). Finally, to assess the proliferative effects of RAGE and RAGEΔICD, proliferation assays using AlamarBlue demonstrated that the RAGE expressing cells were more proliferative (1.3-fold increase) than mock cells under basal conditions. Importantly, C6-RAGEΔICD cells displayed lower proliferative capacity than both mock and RAGE expressing cells (1.5-fold and 2-fold decrease, respectively). Taken together, these data indicate that RAGEΔICD imparts a DN-like-effect on cell adhesion, proliferation and apoptosis.

### RAGEΔICD affects in vitro tumorigenesis

**Figure 7:**
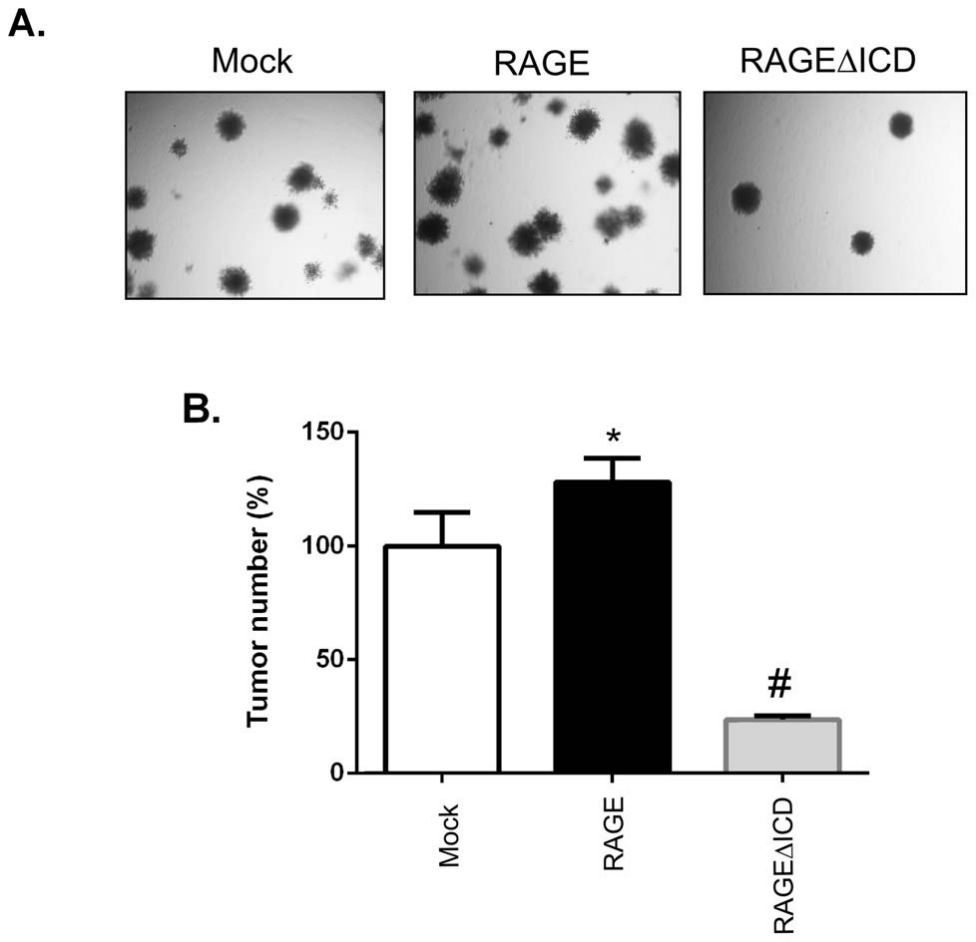
RAGEΔICD inhibits in vitro tumorigenesis. **A.** C6 glioma cells expressing RAGE, RAGEΔICD, or empty vector (mock) were assessed for tumorigenicity using soft agar assays. Cells were seeded in 0.4% agarose and grown for 14 days. Colonies were then stained with crystal violet and visualized on day 14. **B.** Quantification of data shown in “A”. Data are means ± SEM from three independent experiments. *, significant differences (P≤0.05) between mock and RAGE, whereas # significant differences (P≤0.05) between RAGE and RAGEΔICD.

The effect of RAGE on tumorigenesis has been established through blockage of RAGE signaling which abrogates tumor formation [1], [2]. Hence, to address whether RAGEΔICD imparted a DN-like-effect on in vitro tumor formation, we tested the effect of RAGEΔICD on anchorage-independent cell growth in soft agar colony formation assays. RAGE expressing C6 glioma cells increased anchorage-independent cell growth and colony formation compared to mock cells (**Fig. 7**). This finding is consistent with previous studies and confirms that RAGE plays a strong role in tumor formation. Strikingly, RAGEΔICD not only blocked the gain-in-function of cell growth imparted by RAGE, but also it inhibited anchorage-independent growth of cells compared to mock alone (**Fig. 7**). The data indicate RAGEΔICD imparts a DN-like-effect on RAGE induced tumor formation in vitro.

## Discussion

RAGE is a cell surface receptor that mediates diverse cell signaling events via a broad array of ligands. Essential in order to mediate these signaling events is the intracellular or cytoplasmic domain of RAGE. Here, we reported for the first time that an endogenous truncated isoform of RAGE (RAGEΔICD), which lacks the majority of the ICD, is formed through alternative splicing. This novel RAGE isoform is detected in several human and mouse tissues including lung, kidney, brain and heart. We found that expression of RAGEΔICD impaired RAGE-ligand signaling in C6 glioma cells through the MAP kinase pathway. Key cell tumorigenic mechanisms including adhesion, viability, migration and invasion as well as tumor formation were inhibited by RAGEΔICD. Hence, alternative splicing of the RAGE ICD may not only represent a novel mechanism to regulate RAGE signaling, it may also serve as a novel means to control RAGE function.

### The RAGE gene can also undergo alternative splicing to alter its cytoplasmic domain

The alternative splicing of genes is an important mechanism to generate a diversity of protein isoforms from a single gene [48]. The RAGE gene is no exception to this mechanism which leads to an abundance of RAGE products, predominantly at the transcript level [26]–[33]. RAGE alternative splicing mainly results in the production of truncated isoforms lacking the transmembrane domain that are hence secreted by the cell [26]–[33]. We have shown that these soluble isoforms act as RAGE-ligand decoys and as an endogenous mechanism to alter RAGE-ligand signaling [2]. Here, we demonstrate for the first time an alternative splicing of the RAGE ICD which leads to a truncated cytoplasmic domain isoform.

### The novel RAGEΔICD splice variant is expressed in both mouse and human tissues

In our analysis of the distribution of RAGE, RAGEΔICD and other isoforms, we demonstrated that the full-length form of RAGE to be the most prevalent isoform in both human and mice for many tissues (brain, heart, lung, and kidney). The exception to this was in murine kidney, where RAGEΔICD accounted for 86% of all RAGE isoforms detected, and full length RAGE only accounted for 4%. The increased levels of RAGEΔICD seen in kidney is particularly interesting, as RAGE has been shown to play a key role in a number of renal diseases [49] [50]. We thus speculate that as the kidney is exposed to various RAGE ligands under normal physiology compared to other tissues, enhanced expression of RAGEΔICD may be a beneficial mechanism to control RAGE signaling. Nonetheless, in comparison to previous studies [30], [31], we detected a similar distribution of full-length RAGE and other splice variants in all tissues studied which further validate our data. However, it should be noted that our method of screening would not detect all RAGE splicing events as we only studied the exon 8 to 3′UTR region. This therefore includes splice variants affecting exons 1 through 7 which mainly affect the ligand binding site and are not very common. Regardless of this, in our studies here, we were able to detect all prior variants in exons 8 through the 3′UTR in humans and mice as previously described [30], [31].

### RAGEΔICD inhibits RAGE signaling and RAGE-induced cellular functions in a DN-like manner

Given the wide distribution of expression of RAGEΔICD in both humans and mice in tissues, we anticipated that alternative splicing of the ICD of RAGE might play an important role in regulating RAGE-ligand signaling. Truncation/removal of the RAGE ICD by artificial means has been shown to result in a DN-effect that abrogates RAGE signaling and function [1], [13], [18], [20]. This is particularly prominent in tumor models, where DN-RAGE only blocks RAGE-ligand signaling but also tumorigenesis and metastasis in multiple cancers including breast, colorectal, prostate, pancreatic, brain, lung, oral squamous cell, ovarian, as well as lymphoma and melanomas [1], [13], [18], [20] [4], [7]–[12]. We hence examined the role of RAGEΔICD on RAGE signaling in tumor cells using the established C6 glioma model, where RAGE signaling has been shown to have an important role in tumorigenesis [2], [13]. As expected, full-length RAGE over-expression increased RAGE-ligand signaling through the MAP kinase pathway. Furthermore, expression of full-length RAGE increased cellular migration, invasion and adhesion. Contrasting the effects of full-length RAGE, RAGEΔICD blunted the response of cells to RAGE ligands, showing little or no activation of MAP kinase members including ERK1/2, SAPK/JNK and p38. Moreover, in tumorigenic assays, RAGEΔICD decreased cellular migration, invasion and adhesion. These data together demonstrate that RAGEΔICD through truncation of the RAGE cytoplasmic domain affects key signaling properties in tumor cells, which in turn affect mechanisms of tumorigenesis.

A key remaining question is how does the RAGEΔICD change RAGE signaling and function? The protein sequence of the RAGE ICD is highly conserved amongst different species, and our functional studies show the role of RAGEΔICD in RAGE signaling. However, the RAGEΔICD splice variant, unlike the previously engineered DN-RAGE mutants, is not a complete truncation of the RAGE cytoplasmic domain. RAGEΔICD retains the first 10 amino acids common to full-length RAGE before a reading frame shift which produce a truncated protein of 18 amino acids. Importantly, this truncation highlights that different regions of the RAGE ICD may have distinct properties and effects on downstream cellular signaling. In particular, as MAP kinase signaling can result in the activation of numerous different downstream effects including migration, proliferation and apoptosis, this result has implications for RAGEΔICD signaling through different pathways to RAGE.

### Several possibilities may explain the mechanisms by which RAGEΔICD affects RAGE signaling and functions

The RAGE ICD shares no considerable homology with other receptor ICDs and possesses no endogenous receptor tyrosine kinase activity. There are many possibilities how RAGEΔICD may affect RAGE function.

First, RAGEΔICD may alter binding properties with intracellular partners. Several studies have identified a number of candidates capable of binding to the cytoplasmic domain of RAGE including ERK1/2, PKCζ, DOCK7, TIRAP, and diaphanous-1 [13], [21]–[23,23]. The latter, which was identified to bind to the R366/Q367 region of the RAGE ICD, is the most studied to-date of these factors [51]. Because R366/Q367 region is conserved between RAGE/RAGEΔICD, diaphanous-1 may not be mediating the effects seen in this study. Furthermore, for all other binding partners for the RAGE ICD, it has not been shown where they interact with this domain of RAGE, or whether the interaction is direct. However, as we did not test the interaction of any of these binding partners identified for RAGEΔICD in this study, we can only speculate about these conclusions. It is also possible that the effects seen with RAGEΔICD may be mediated by a novel interacting partner, that is distinct from that for canonical RAGE.

Second, RAGEΔICD may affect the phosphorylation of the RAGE cytoplasmic domain. Recently, it was demonstrated that S391 of RAGE is phosphorylated by PKCζ [22], a region that is missing in the RAGEΔICD. This is of obvious interest because the function of this phosphorylation site has not been clearly demonstrated.

Finally, the changes in the cytoplasmic domain of RAGEΔICD may affect the overall conformation of this novel isoform. This could possibly explain that although the diaphanous-1 binding site may still exist in the ICD of RAGEΔICD, altered protein folding could prevent its binding. These changes in conformation of RAGEΔICD could also affect RAGE dimerization which has been shown to have a role in signaling and function [52]–[54]. However, contradictory studies exist as to whether the intra- or extra –cellular domains are more important to mediate dimerization of RAGE at the cell surface [52]–[54]. Further, it is possible that the altered sequence of RAGEΔICD could affect the overall conformation of the protein and affect ligand binding and/or affinity. However, numerous studies have shown that alteration of the extracellular domain and not TM or ICD affect RAGE-ligand affinity [46] [13] [55] [56] [57] and therefore making this possibility less likely.

### Limitations of the current study

Whilst RAGE signalling is induced by a wide range of ligands, we only tested S100B which is the most extensively characterized RAGE ligand in functional tumor cell studies. As a further test of RAGE function in many assays, we did not solely test specific RAGE ligands but tested cells under serum stimulation or normal physiological growth conditions. Also, RAGE signalling has been shown to be as diverse as its ligand family and extends beyond the MAP kinase pathway tested here. Our rationale for testing this pathway was that in our cell model, we have previously shown MAP kinase in particular JNK to be the prominent pathways for regulating tumorigenesis [2]. Therefore, future studies would be required to test the role of other ligands and signaling pathways to determine how RAGEΔICD affects RAGE function. It is possible that RAGEΔICD, due to its sequence difference with full-length RAGE, may signal through a different pathway(s) to mediate the inhibitory effects seen in the current study. Another limitation of our study is that in the first description of this novel RAGEΔICD isoform, we have limited our studies to expression of this variant at the cDNA level and tested its role in cells by overexpression. Whilst this may be an accepted first approach to properly validate this variant detection of RAGEΔICD at the protein level in tissue/cells, endogenous detection at the protein level would be needed. As RAGEΔICD differs only from RAGE by 4 bp at the RNA level, this makes it impossible to study the role of RAGEΔICD in cells by siRNA approaches or by designing primer/probe sets for QPCR analysis. Our analysis of RAGEΔICD in tissue cDNA from both human and mouse samples would suggest biological relevance as this variant is conserved amongst these species. Furthermore, bioinformatics analysis would suggest that this is not a splice variant targeted for nonsense-mediated mRNA decay as for numerous other RAGE splice variants [30] [31]. Therefore, future studies are clearly needed with specific antibodies raised against RAGEΔICD to verify its expression at the cell and tissue level endogenously and to assess whether its levels change under pathological states.

In conclusion, our data reveal for the first time, the occurrence of a truncated cytoplasmic domain splice variant of RAGE. We demonstrate that RAGEΔICD displays differential signaling and cellular effects to RAGE and therefore has obvious implications in pathogenic scenarios where RAGE has been implicated including cancer, vascular disease and diabetes. The data therefore suggest that alternative splicing of the RAGE ICD may be a novel means by which to regulate RAGE signaling and as a therapeutic target in RAGE mediated pathogenic states including cancer.

## Supporting Information

Figure S1 Detection of mouse RAGE alternative splice variants. **A**. Exon and restriction map of the region amplified for analysis for full-length mouse RAGE cDNA. Primer sites used to amplify the RAGE exon 8 to 3′UTR region are indicated by arrows above the exons/cDNA. **B**. A region is amplified from exon 8 to the 3′UTR of RAGE and digested by HpyAV. The splice variation of RAGEΔICD (mRAGE_v20) results in the loss of an HpyAV site (bold arrow). Resulting DNA fragments are shown in base pairs. The splice site affected by RAGEΔICD is shown by a bold arrow. **C**. PCR product of the RAGE exon 8 to 3 UTR amplification for splice variants detected is shown. **D**. Restrictive digestion of the mouse RAGE cDNA PCR products with HpyAV. The corresponding splice variant classification is shown above the digestion. DNA fragments were sized against a 1-kb DNA ladder as indicated on each gel.
(DOCX)

Figure S2 Detection of human RAGE alternative splice variants. **A.** Exon and restriction map of the region amplified for analysis for full-length human RAGE cDNA. Primer sites used to amplify the human RAGE exon 8 to 3′UTR region are indicated by arrows above the exons/cDNA. **B**. A region is amplified from exon 8 to the 3′UTR of RAGE and digested by HpyAV and Bam HI. The splice variation of RAGEΔICD (RAGE_v20) results in the loss of the HpyAV site (bold arrow). Resulting DNA fragments are shown in base pairs. The splice site affected by RAGEΔICD is shown by a bold arrow. **C**. PCR product of the RAGE exon 8 to 3 UTR amplification for splice variants detected is shown. **D**. Restrictive digestion of the human RAGE cDNA PCR products with HpyAV. The corresponding splice variant classification is shown above the digestion. DNA fragments were sized against a 1-kb DNA ladder as indicated on each gel.
(DOCX)
