# Supplementary material for: Alternative Splicing of the RAGE Cytoplasmic Domain Regulates Cell Signaling and Function
Source: PLoS One. 2013 Nov 8;8(11):e78267. doi: 10.1371/journal.pone.0078267 (PMC3832623; doi:10.1371/journal.pone.0078267)
Supplement: Figure S1 — Detection of mouse RAGE alternative splice variants. A. Exon and restriction map of the region amplified for analysis for full-length mouse RAGE cDNA. Primer sites used to amplify the RAGE exon 8 to 3′UTR region are indicated by arrows above the exons/cDNA. B. A region is amplified from exon 8 to the 3′UTR of RAGE and digested by HpyAV. The splice variation of RAGEΔICD (mRAGE_v20) results in the loss of an HpyAV site (bold arrow). Resulting DNA fragments are shown in base pairs. The splice site affected by RAGEΔICD is shown by a bold arrow. C. PCR product of the RAGE exon 8 to 3 UTR amplification for splice variants detected is shown. D. Restrictive digestion of the mouse RAGE cDNA PCR products with HpyAV. The corresponding splice variant classification is shown above the digestion. DNA fragments were sized against a 1-kb DNA ladder as indicated on each gel. (DOCX) [file pone.0078267.s001.docx]

**Alternative splicing of the RAGE cytoplasmic domain regulates cell signaling and function**

Joel Jules^1^, Dony Maiguel^2^, Barry I Hudson^1^

**
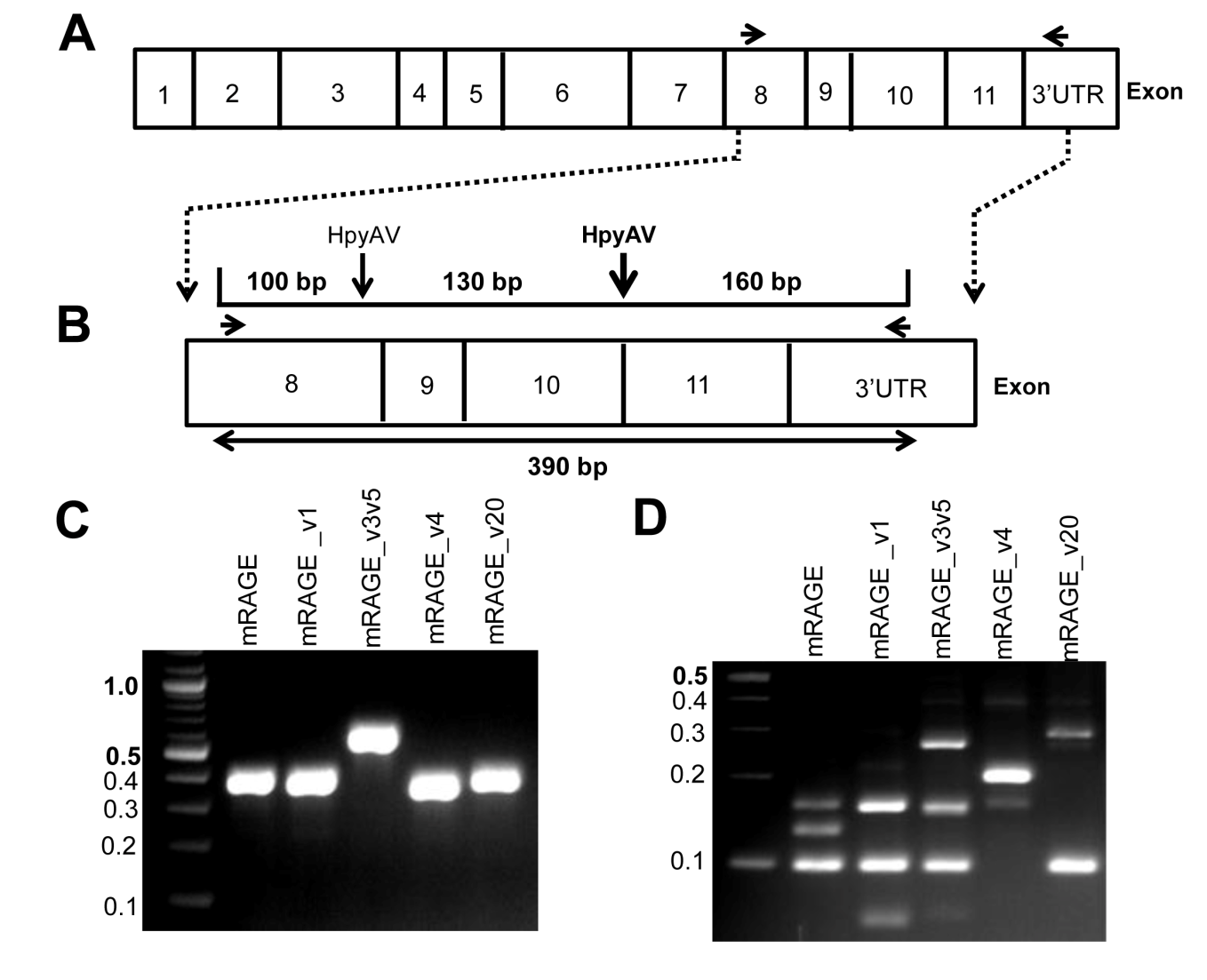
Fig. S1. Detection of mouse RAGE alternative splice variants.** **A**. Exon and restriction map of the region amplified for analysis for full-length mouse RAGE cDNA. Primer sites used to amplify the RAGE exon 8 to 3’UTR region are indicated by arrows above the exons/cDNA. **B.** A region is amplified from exon 8 to the 3’UTR of RAGE and digested by HpyAV. The splice variation of RAGEΔICD (mRAGE_v20) results in the loss of an HpyAV site (bold arrow). Resulting DNA fragments are shown in base pairs. The splice site affected by RAGEΔICD is shown by a bold arrow. **C**. PCR product of the RAGE exon 8 to 3 UTR amplification for splice variants detected is shown. **D**. Restrictive digestion of the mouse RAGE cDNA PCR products with HpyAV. The corresponding splice variant classification is shown above the digestion. DNA fragments were sized against a 1-kb DNA ladder as indicated on each gel.
