# Supplementary material for: Alternative Splicing of the RAGE Cytoplasmic Domain Regulates Cell Signaling and Function
Source: PLoS One. 2013 Nov 8;8(11):e78267. doi: 10.1371/journal.pone.0078267 (PMC3832623; doi:10.1371/journal.pone.0078267)
Supplement: Figure S2 — Detection of human RAGE alternative splice variants. A. Exon and restriction map of the region amplified for analysis for full-length human RAGE cDNA. Primer sites used to amplify the human RAGE exon 8 to 3′UTR region are indicated by arrows above the exons/cDNA. B. A region is amplified from exon 8 to the 3′UTR of RAGE and digested by HpyAV and Bam HI. The splice variation of RAGEΔICD (RAGE_v20) results in the loss of the HpyAV site (bold arrow). Resulting DNA fragments are shown in base pairs. The splice site affected by RAGEΔICD is shown by a bold arrow. C. PCR product of the RAGE exon 8 to 3 UTR amplification for splice variants detected is shown. D. Restrictive digestion of the human RAGE cDNA PCR products with HpyAV. The corresponding splice variant classification is shown above the digestion. DNA fragments were sized against a 1-kb DNA ladder as indicated on each gel. (DOCX) [file pone.0078267.s002.docx]

**Alternative splicing of the RAGE cytoplasmic domain regulates cell signaling and function**

Joel Jules^1^, Dony Maiguel^2^, Barry I Hudson^1^

**357bp**

1

3

4

5

6

7

8

10

11

3’UTR

**A**

**Exon**

BamHI

8

9

10

11

3’UTR

**HpyAV**

**125bp**

**76bp**

**Exon**

**156bp**

2

9

0.1

0.2

0.3

0.4

**0.5**

**1.0**

**B**

RAGE

RAGE _v1

RAGE _v20


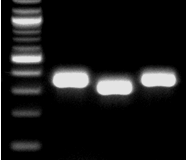


**D**

0.1

0.2

0.3

**0.5**

RAGE

RAGE _v1


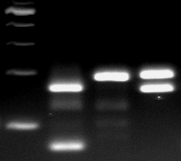


RAGE _v20

**Fig. S2. Detection of human RAGE alternative splice variants. A.** Exon and restriction map of the region amplified for analysis for full-length human RAGE cDNA. Primer sites used to amplify the human RAGE exon 8 to 3’UTR region are indicated by arrows above the exons/cDNA. **B.** A region is amplified from exon 8 to the 3’UTR of RAGE and digested by HpyAV and Bam HI. The splice variation of RAGEΔICD (RAGE_v20) results in the loss of the HpyAV site (bold arrow). Resulting DNA fragments are shown in base pairs. The splice site affected by RAGEΔICD is shown by a bold arrow. **C**. PCR product of the RAGE exon 8 to 3 UTR amplification for splice variants detected is shown. **D**. Restrictive digestion of the human RAGE cDNA PCR products with HpyAV. The corresponding splice variant classification is shown above the digestion. DNA fragments were sized against a 1-kb DNA ladder as indicated on each gel.
